# Supplementary material for: Psychological Impact of Exposure to the COVID-19 Sanitary Crisis on French Healthcare Workers: Risk Factors and Coping Strategies
Source: Front Psychiatry. 2021 Nov 18;12:701127. doi: 10.3389/fpsyt.2021.701127 (PMC8637162; doi:10.3389/fpsyt.2021.701127)
Supplement: Supplementary file 1 [file Presentation_1.pdf]

Supplementary Material 1. Visual analogic scales measuring the effect of COVID-19 on mental health.

Please answer from 0 (minimum) to 10 (maximum)

**Theme**	**Questions**
Worries for close relations' health	I worry for my close relations' health
Fear of contaminate a close relation	I am scared of contaminating a close relation.
Worries for children's health	I worry for my children's health
Fear of contaminating my children	I am scared of contaminating my children.
Worries about watching children	I worry about my children watch.
Powerlessness at work	I feel powerless on my professional life (e.g. for my patients care)
Powerlessness in the personal life	I feel powerless on my personal life (e.g. for protection close relations).
Ethical dilemmas	I had to make ethical dilemmas (e.g. choosing which patients to prioritize).
Not answering patients demands	I had the feeling of not responding to my patients' demands.
Lack of material	I missed protecting material at work.
Setting close relation in danger	I feel guilty at my work about the crisis.
Guilty at work	I feel guilty at work.
Anger at work	I feel angry at my work about the crisis.
Increase anxiety	Since the beginning of the crisis, I feel anxious.
Increase sadness	Since the beginning of the crisis, I feel sad.
Sleep disturbance	Since the beginning of the crisis, my sleep is significantly disturbed.
Appetite disturbance	Since the beginning of the crisis, my appetite is significantly disturbed.
Feeling of regret	I strongly feel regret.
Increase mistakes	I have the feeling of making more mistakes since the beginning of the crisis.
Putting patients in danger	I think I put my patients' life in danger.
Respect behavior toward patients	I think I behave respectfully toward my patients.
Exemplary behavior from colleagues	I think my colleagues' behavior has been exemplary.
Good reaction from the hospital	I think the hospital as reacted as best as possible.
Good material management from the hospital	I think the hospital has well managed material.
Delay for information from state	I blame the state for the delay for information.
Poor management of the crisis from state	I think the state did not manage the crisis as well as possible.
Non respect of quarantine	I think citizens do not respect the instructions.
Guilty in the personal life	I feel guilty in my personal life about the crisis.
Self-blame for non-protection of close relations	I blame myself for not being able to protect my close relations/my children.
Blaming the state for lack of help for healthcare workers	I blame the state for doing nothing to help healthcare workers.
